# CT and MRI in the Evaluation of Thoracic Aortic Diseases

**DOI:** 10.1155/2013/797189

**Published:** 2013-12-11

**Authors:** Prabhakar Rajiah

**Affiliations:** Cardiothoracic Imaging, University Hospitals of Cleveland, Case Medical Center, Case Western Reserve University School of Medicine, 111000 Euclid Avenue, Cleveland, OH 44106, USA

## Abstract

Computed tomography (CT) and magnetic resonance imaging (MRI) are the most commonly used imaging examinations to evaluate thoracic aortic diseases because of their high spatial and temporal resolutions, large fields of view, and multiplanar imaging reconstruction capabilities. CT and MRI play an important role not only in the diagnosis of thoracic aortic disease but also in the preoperative assessment and followup after treatment. In this review, the CT and MRI appearances of various acquired thoracic aortic conditions are described and illustrated.

## 1. Introduction

Computed tomography (CT) and magnetic resonance imaging (MRI) are the commonly used imaging examinations for evaluating a variety of acquired aortic lesions including aneurysm, dissection, ulcer, intramural hematoma, traumatic injury, and aortitis. Both CT and MRI have excellent spatial and temporal resolutions, wide fields of view, and multiplanar imaging capabilities [1].

Multidetector-row CT (MDCT) of the aorta is more commonly used than MRI because of the rapid scan times and wider availability. CT is usually performed with electrocardiographic (ECG) gating in order to avoid motion artifacts in the ascending aorta that might produce a false appearance of dissection or result in inaccurate measurements [2]. Since ECG gating is associated with significant increase in radiation dose, various dose reduction techniques such as prospective ECG triggering, ECG-based tube current modulation, automatic exposure control, lower peak kilovoltage (kVp), and iterative reconstruction algorithms are used [3–6]. CT requires the use of potentially nephrotoxic iodinated contrast agents, which should be avoided in patients with renal dysfunction. Serum creatinine and GFR should be obtained in patients receiving intravenous contrast. If the GFR is >45 mL/min, contrast can be administered without any preparation. If the GFR is between 30 and 44 mL/min, contrast can be administered with pre- and postprocedural hydration. If GFR is less than 30 mL/min, contrast should not be administered. If the patient is on dialysis, contrast can be administered regardless of GFR. MRI does not involve the use of radiation or nephrotoxic iodinated contrast media. Three-dimensional contrast-enhanced magnetic resonance angiogram (3D CE-MRA) acquired after administration of gadolinium can be reconstructed in multiple planes enabling evaluation of complex vascular abnormalities. These examinations are typically acquired without ECG gating, which can lead to blurring of the aortic root and proximal ascending aorta. However, ECG-gated 3D CE-MRA is technically challenging and is not completely devoid of blurring. Gadolinium is avoided in patients with severe renal dysfunction due to the risk of nephrogenic systemic fibrosis and in pregnant patients. Images with similar quality can be acquired using a noncontrast, free breathing navigator-gated 3D steady state free precession (SSFP) MRA in which images are acquired only in diastole and during expiration [7]. Cine SSFP sequences can be used to quantify cardiac volumes and function and evaluate dynamic changes in the aorta and associated cardiac malformations. Velocity encoded phase contrast (PC-MRI) is used to quantify valvular abnormalities and estimate the hemodynamic significance of obstructive lesions. A T2-weighted black blood inversion recovery fast spin echo (FSE) sequence can be used to evaluate the aortic wall and a short tau inversion recovery (STIR) sequence can demonstrate edema in the aortic wall.

## 2. Normal Thoracic Aorta 

**Figure 1:**
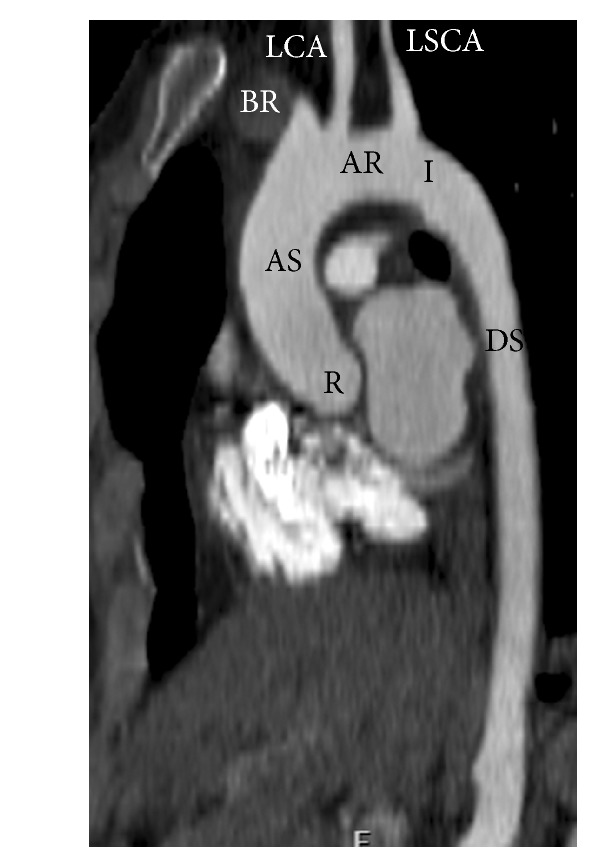
Normal  thoracic  aorta—sagittal reconstructed CT image of thoracic aorta showing the various segments of thoracic aorta and the arch branch vessels. R: root; AS: ascending aorta; AR: arch; I: isthmus; DS: descending thoracic aorta. BR: brachiocephalic artery; LCA: left common carotid artery; LSCA: left subclavian artery.

The thoracic aorta can be divided into several segments, namely, the root, ascending aorta, arch, and descending aorta (Figure 1). The root has three components: the annulus, the sinuses of Valsalva, and the sinotubular junction. The coronary arteries originate from the sinuses adjacent to the sinotubular junction. The ascending aorta extends from the sinotubular junction to the origin of brachiocephalic artery. The arch extends from the brachiocephalic artery origin to the left subclavian artery origin and is further subdivided into proximal and distal segments. The proximal arch extends from brachiocephalic origin to the left common carotid origin. The distal arch extends from the left common carotid origin to the left subclavian artery. The descending thoracic aorta extends from the ligamentum arteriosum to the level of the diaphragmatic hiatus. The most proximal portion of the descending thoracic aorta appears slightly dilated and is called the “aortic spindle.”

## 3. Aortic Measurements

**Figure 2:**
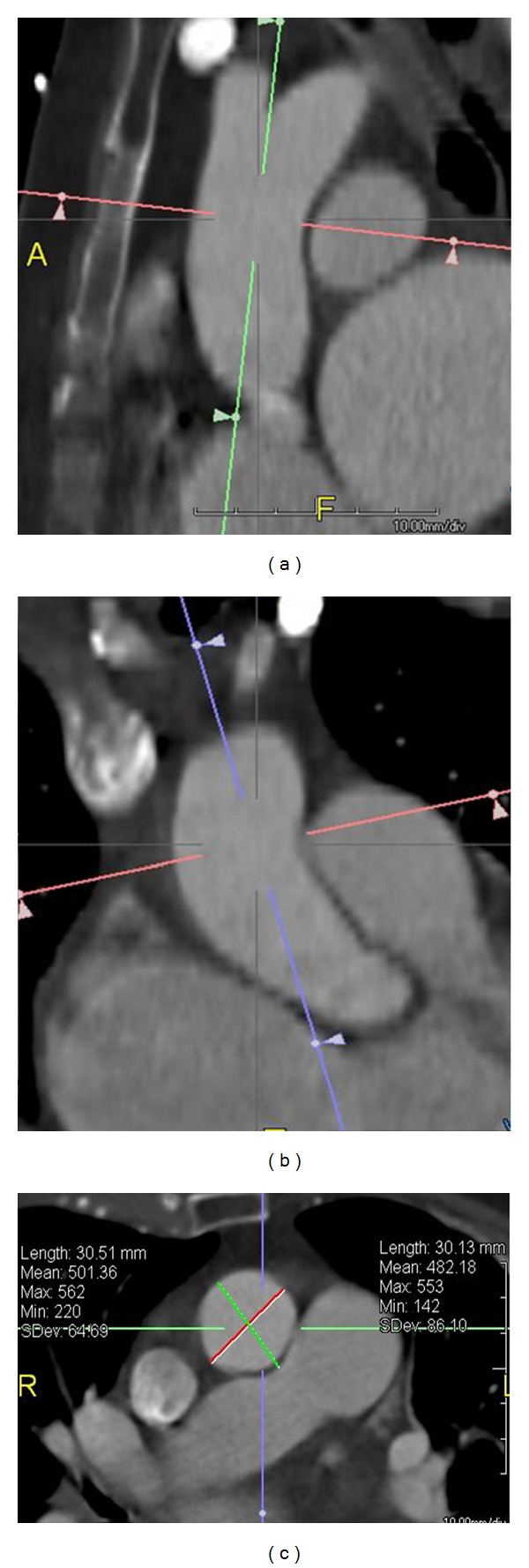
Measurement  of  aortic  size  by  CT  scan. Double oblique short axis image of mid ascending aorta (c) is acquired from orthogonal coronal (a) and sagittal (b) images at the level of mid ascending aorta. From the double oblique short axis image, minimum and maximum diameters and area are measured.

Accurate measurement of the aorta is vital in followup of thoracic aortic aneurysms and determining suitability for surgical or endovascular repair. Aortic measurements are made in true short axis projection acquired from double oblique views, from one blood-wall boundary to the other. With CT, ECG gating is essential for accurate measurement, particularly in the ascending aorta (Figure 2).

**Figure 3:**
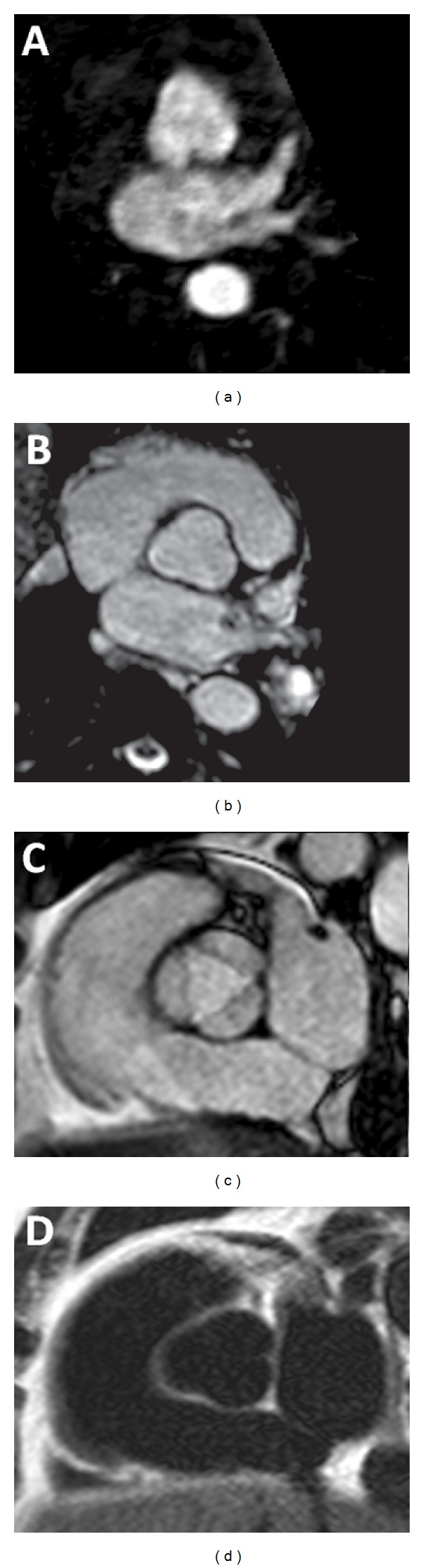
Measurement of the aortic size in various MRI sequences; (a) 3D contrast enhanced MR angiography. The margins are blurred since the acquisition is not ECG gated. (b) Noncontrast 3D steady state free precession (SSFP) image has sharp margins due to ECG gating. (c) 2D SSFP cine image, which can be acquired throughout cardiac cycle, enabling measurement in systole or diastole. (d) T2-weighted black blood image, which is also acquired with ECG gating.

On MRI, various sequences are used for measurement, including 3D CE-MRA, noncontrast 3D SSFP, 2D cine SSFP, and 2D black blood T2-weighted images (T2 BB) (Figure 3). Of these, the 3D navigator SSFP sequence is the optimal choice for diameter measurement because of ease of acquisition, edge profile, and good interobserver correlation. Measurements are higher for 3D SSFP and 3D CE MRA and smallest for T2 BB. Interobserver agreement is best for T2 BB and worst for 3D CE-MRA. Absence of ECG gating results in poor edge sharpness for 3D CE-MRA images [8]. With SSFP images of the aortic root, the cusp-commissure measurement is smaller by 2-3 mm than diastolic cusp-cusp measurements. However, this measurement better correlates to age, body surface area, and echocardiographic root measurement [9]. The average of three measurements is usually used, except with an asymmetric root, for which all the three measurements should be provided. Maximal intensity projections (MIPs) should not be used for measurement since they show only the lumen and result in underestimation.

## 4. Aortic Dilation

**Figure 4:**
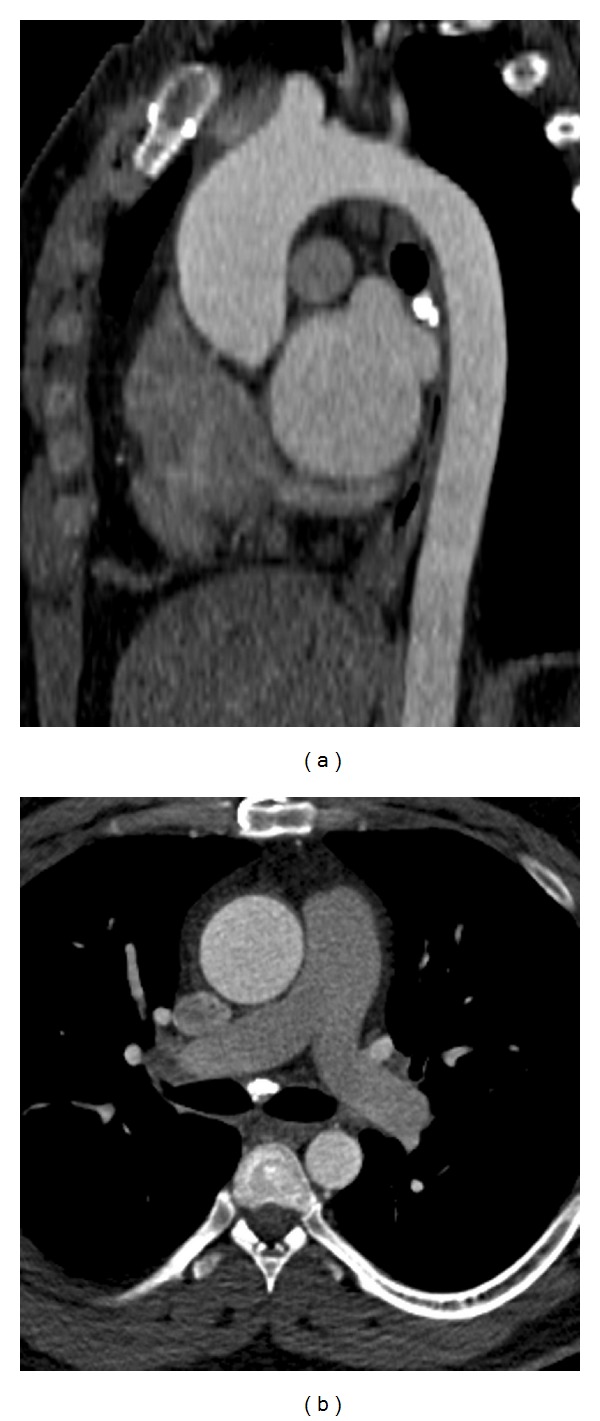
Aortic dilation—(a) sagittal oblique reconstructed CT in a 41-year-old male shows dilated ascending aorta (4.6 cm) not having reached the size required for a diagnosis of aneurysm. (b) Axial CT image shows the dilated ascending aorta but normal descending thoracic aorta.

The normal aortic annulus measures 26.3 mm ± 2.8 mm in the coronal plane and 23.5 ± 2.7 mm in the sagittal plane with no significant change between systole and diastole [10]. The normal ascending aorta usually measures 3.6 cm in females and 3.8 cm in males [11]. The proximal descending aorta is considered enlarged when it exceeds 2.6 cm and the distal descending aorta is considered enlarged when it exceeds 2.4 cm [11]. However, for simplicity, the ascending aorta is considered enlarged when it is larger than 4 cm and the descending aorta when it is larger than 3 cm (Figure 4). An aneurysm is diagnosed when the ascending aorta is larger than 5 cm and the descending aorta is larger than 4 cm [11]. *z* scores are used to diagnose aortic dilations and aneurysms, particularly in children. A *z* score represents standard deviation from the mean aortic diameter, normalized for the patient's age and body surface area [12, 13]. Aortic root dilation is associated with risk factors of coronary artery disease and predicts cardiac failure, stroke, and all causes of cardiovascular mortality [9].

## 5. Aneurysm

**Figure 5:**
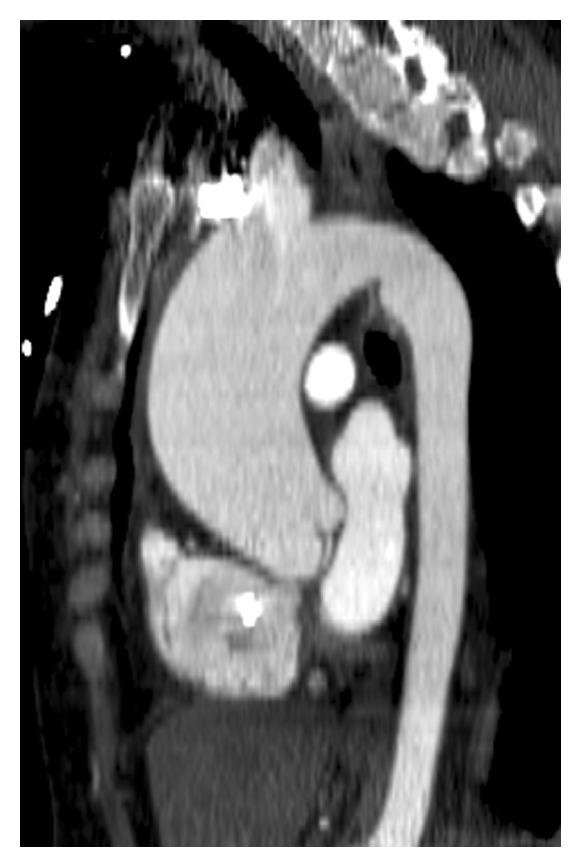
Fusiform ascending aortic aneurysm—sagittal reconstructed CT image in a 47-year-old male shows a smooth, fusiform aneurysm of the ascending aorta, which tapers to normal caliber at the proximal arch.

An aneurysm is defined as permanent localized dilation of the aorta, either 50% or 2 standard deviations larger than normal expected diameter for that aortic segment (Figure 5). Atherosclerosis is the overall most common cause of aneurysm, accounting for 70% of these cases. Medial degeneration is the most common cause of ascending aortic aneurysm and atherosclerosis is the most common cause of descending thoracic aneurysm. Atherosclerotic aneurysms most commonly occur in the descending thoracic aorta. Most of these aneurysms present in the sixth and seventh decades of life, with a male predominance and involvement of abdominal aorta in one-third of patients. Sixty percent of patients have systemic hypertension, which can result in cystic medial degeneration. Patients with aortic aneurysms can be asymptomatic or present with chest pain or discomfort. Other causes of aortic aneurysm include aortic dissection, Marfan syndrome, congenital medial degeneration, bicuspid aortic valve, coarctation, Ehlers-Danlos syndrome, Loeys-Dietz syndrome, syphilis and other infections, arteritis, or trauma. Less common causes include ankylosing spondylitis, rheumatoid arthritis, rheumatic fever, systemic lupus erythematosus, scleroderma, Behçet disease, psoriasis, ulcerative colitis, Reiter syndrome, and radiation therapy [14].

Syphilitic aneurysms are rare, seen in 12% of these patients, with a latency of 10–30 years, most commonly in the ascending aorta, with high risk for rupture and less risk for dissection [14]. With bicuspid valve, aortic dilation results from an intrinsic aortopathy and not as a result of valvular stenosis. Aortitis associated with ankylosing spondylitis, rheumatoid arthritis, giant cell arteritis, and relapsing polychondritis affects the ascending aorta. With rheumatic fever, aortic involvement is segmental or total. Takayasu arteritis commonly affects the aortic arch and branches. Postoperative aneurysms can be seen at suture lines or at site of cannulation, aortotomy, needle puncture, or cannulation. Traumatic pseudoaneurysm most commonly occurs at the isthmus (90%) and less commonly in the ascending aorta or descending aorta near the diaphragmatic hiatus [14].

**Figure 6:**
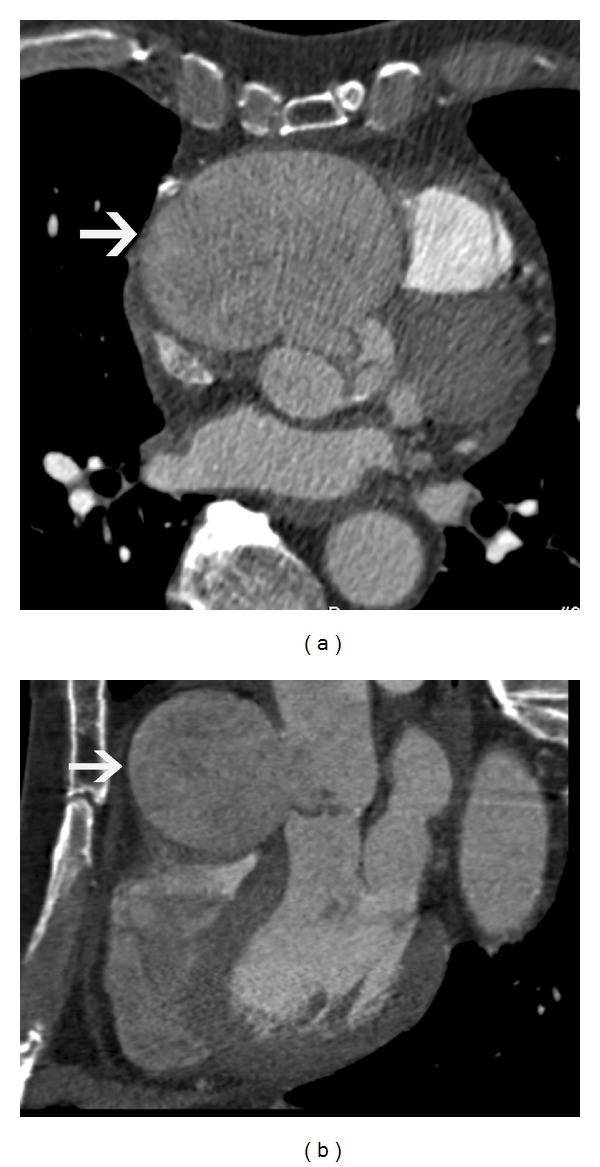
Saccular  aortic  root  aneurysm—(a) short axis reconstructed CT image showing a giant aneurysm of the aortic root, originating from the right sinus of Valsalva. (b) Coronal reconstructed CT image shows the saccular aneurysm with narrow neck originating from the right sinus of Valsalva.

**Figure 7:**
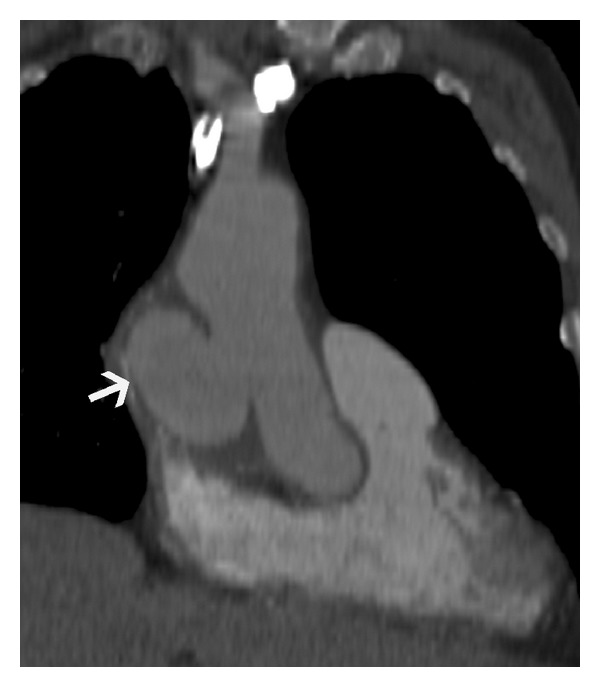
Pseudoaneurysm—coronal reconstructed CT image in a 53-year-old male following aortic surgery shows a pseudoaneurysm with narrow neck originating from the lateral aspect of the ascending aorta.

Thoracoabdominal aneurysms can be classified based on location using the modified Crawford classification system: type  I—from level of left subclavian origin to above the renal arteries; type  II—from the left subclavian artery origin to below the renal arteries; type  III—from the level of sixth intercostal space to below renal arteries; type  IV—from level of 12th intercostal space to below the renal arteries (total abdominal aortic aneurysm); and type  V—from level of sixth intercostal space to above the renal arteries [15]. Aneurysms are most common in the ascending segment (60%), followed by descending segment (40%) and arch (10%). Both thoracic and abdominal segments are affected in 10% of patients with aortic aneurysm [15]. Aneurysms are also classified as either fusiform or saccular (Figure 6). A fusiform aneurysm has symmetrical dilation involving the entire circumference of the aortic wall, while a saccular aneurysm is more localized with outpouching of one portion of the aortic wall. True aneurysms involve all the three mural layers, are usually fusiform [11], and are most commonly caused by atherosclerosis [14]. All the three mural layers are not involved with pseudoaneurysms (false aneurysms), which are often associated with intimal and, occasionally, medial disruption, with containment of blood by adventitial and periadventitial tissues. Pseudoaneurysms are usually saccular (Figure 7) and are most commonly the result of trauma, infection, instrumentation, or penetrating atherosclerotic ulcers.

CT and MRI clearly characterize aortic aneurysms and are used for assessing size. On spin-echo MRI images, the lumen has signal void and thrombus has intermediate signal intensity. With MRA, particularly on MIPs, only the lumen is seen, and the aneurysm size is underestimated. CT clearly shows mural calcification and low-attenuation crescenteric or concentric thrombus with irregular margins and contiguous interface with aortic lumen.

Large aneurysms can compress the adjacent structures such as the superior vena cava (SVC), recurrent laryngeal nerve, esophagus, or coronary arteries. Dissection and rupture are other complications. The risk of rupture increases with aneurysm size, and outcomes are better when surgical repair is performed electively. Generally, aneurysms are repaired when the ascending aorta exceeds 5.5 cm, the descending aorta exceeds 6 cm, or if the aneurysm enlarges more than 1 cm per year. In patients with Marfan syndrome, surgery is usually performed when the ascending aorta exceeds 5 cm, and repair is undertaken in patients with Loeys-Dietz syndrome when the ascending aorta exceeds 4.5 cm.

**Figure 8:**
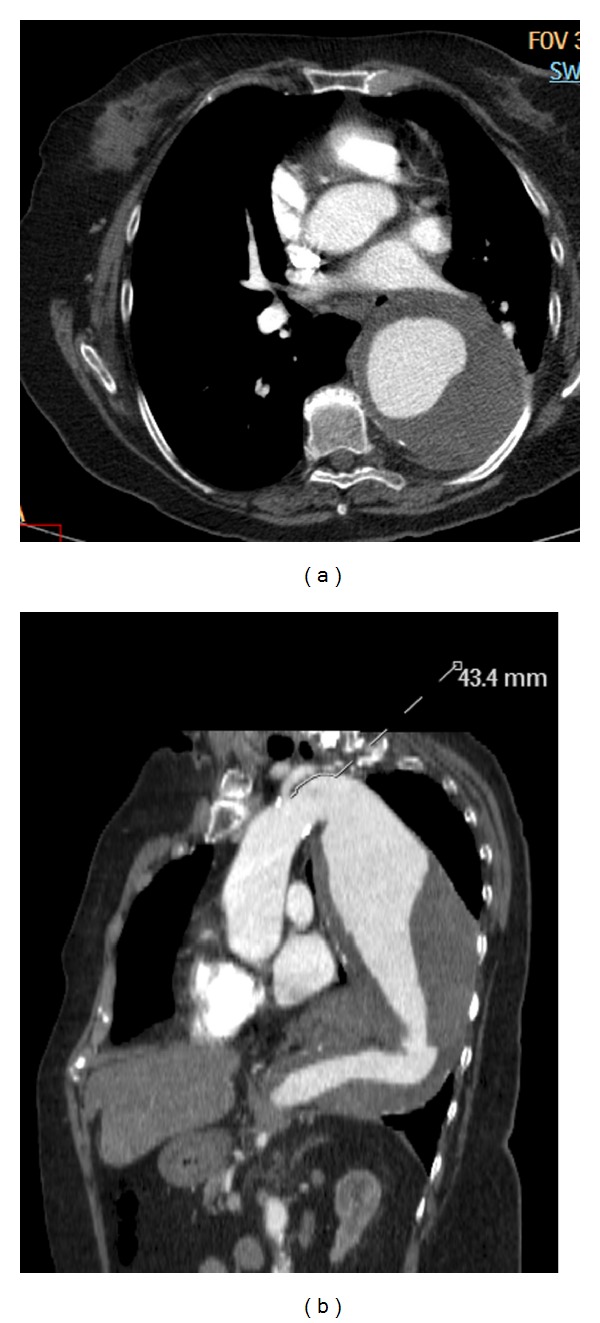
Descending  thoracic  aortic  aneurysm—(a) axial CT scan image shows a large aneurysm of the descending thoracic aorta, with large intraluminal thrombus. (b) Oblique reconstructed CT image shows a large fusiform descending thoracic aortic aneurysm. There is a 4.3 cm distance between the neck of the fusiform aneurysm and the left subclavian artery, which makes this patient suitable for endovascular repair.

CT scan is used for determining suitability for endovascular repair of descending thoracic aortic aneurysms. The criteria for endovascular repair include at least 15 mm distance between the aneurysm and the origin of the left subclavian artery or celiac artery (Figure 8); maximum aortic diameter in landing zone of 4 cm; and absence of thrombus or atheroma in the landing zone [14].

## 6. Annuloaortic Ectasia

**Figure 9:**
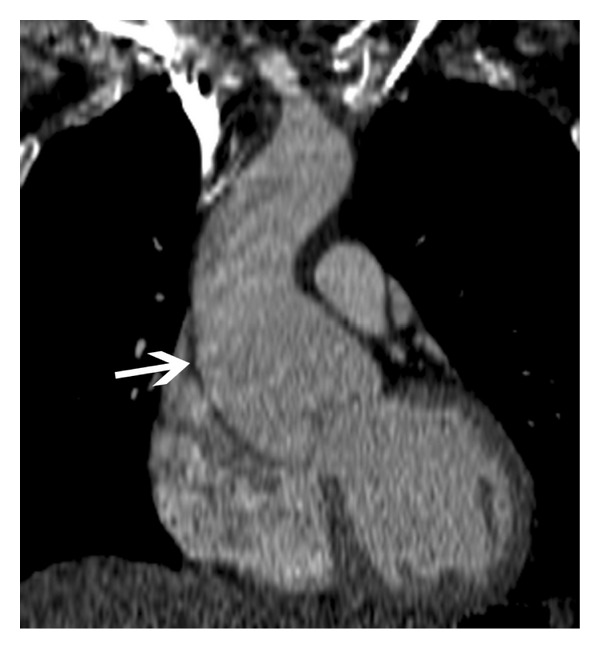
Annuloaortic ectasia—coronal oblique MRI image in a 30-year-old male with Marfan syndrome shows dilated aortic root and ascending aorta with effacement of the sinotubular junction (arrow) consistent with annuloaortic ectasia.

Annuloaortic ectasia is symmetric dilation of the aortic root and ascending aorta with effacement of the sinotubular junction. The ascending aorta has a pear shaped appearance and the arch is of normal caliber (Figure 9). It most commonly occurs with Marfan syndrome, but it can also be seen in other conditions such as Ehlers-Danlos syndrome, homocystinuria and osteogenesis imperfecta or be idiopathic. Ascending aortic aneurysm is also seen in syphilis, bicuspid aortic valve, aortitis, and postoperative patients.

## 7. Traumatic Aortic Injury

Aortic injury is a serious sequela of blunt chest trauma. The most common site for aortic injury is in the ascending aorta; however, most patients die at the scene of injury from cardiac tamponade. The most common location of injury in patients undergoing imaging is the arch at or near the level of ligamentum arteriosum, either from rapid deceleration resulting in forward movement of the distal arch with a stationary descending aorta anchored by the ligamentum arteriosum and intercostal vessels or impingement of the aorta by the sternum and ipsilateral first rib and clavicle during anteroposterior compression of the chest [14].

**Figure 10:**
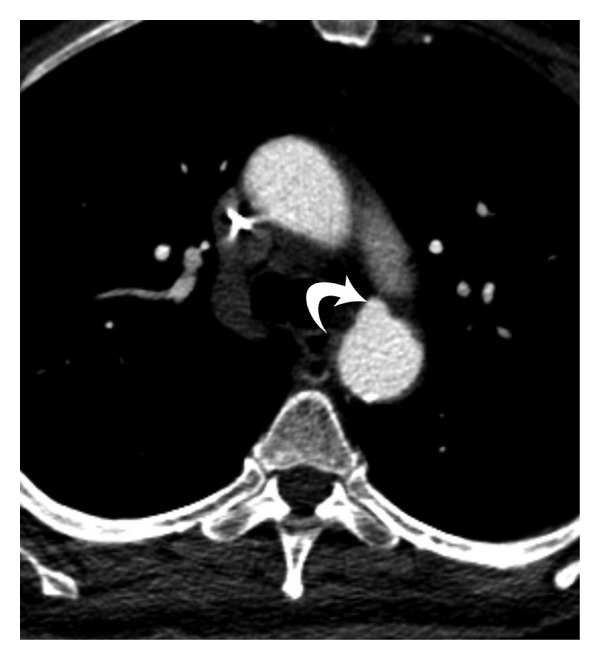
Acute  traumatic  aneurysm—postcontrast axial CT scan image in a 28-year-old male who was involved in a motor vehicle accident shows a small focal outpouching from the anterior aspect of the proximal descending thoracic aorta (curved arrow), which is indicative of aortic trauma. Note the absence of mediastinal hematoma in this patient.

CT is the most commonly used examination for evaluation of the aorta in the setting of trauma and is highly sensitive and specific. Direct CT findings of acute aortic injury include deformity of the aortic contour (Figure 10), intimal flap, intraluminal debris, pseudoaneurysm, and intramural hematoma [16]. Pseudoaneurysm and intramural hematoma are the most common findings [17]. Active extravasation is rarely seen. Mediastinal hematoma is not specific for aortic injury and may be absent in 9–22% of patients with confirmed traumatic aortic injury [17]. CT is more sensitive than angiography in the detection of subtle aortic injuries [17]. Aortic transection accounts for 16% of deaths from motor vehicle accidents [16].

**Figure 11:**
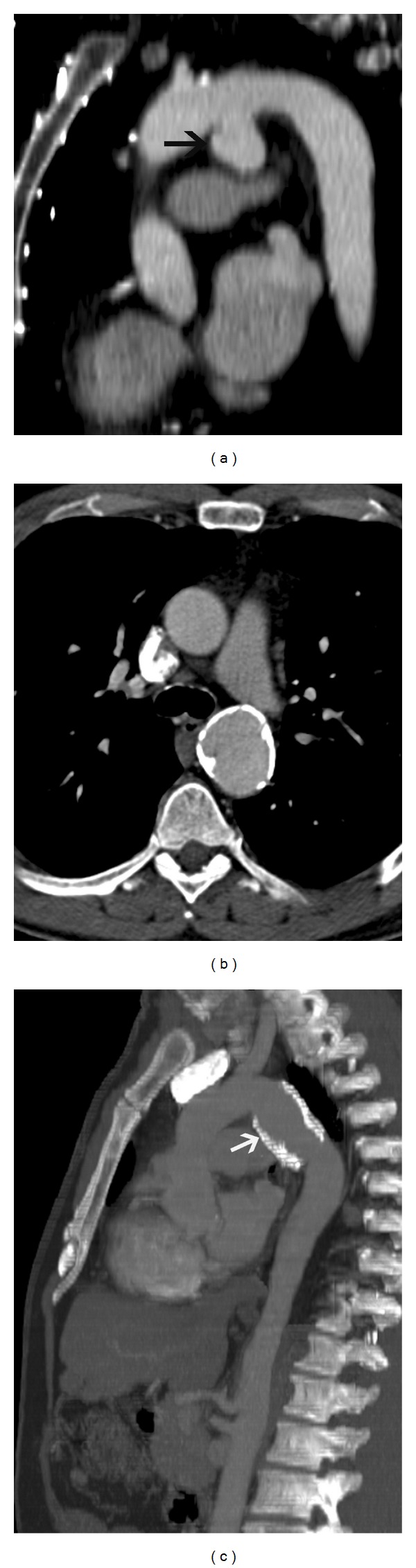
Chronic traumatic aneurysm—(a) sagittal reconstructed CT image in a 71-year-old male involved in road traffic accident five years back shows a saccular pseudoaneurysm in the aortic isthmus. (b) Axial CT image in a 65-year-old male involved in motor vehicle accident ten years back shows a calcified aneurysm of the aortic isthmus. (c) Sagittal reconstructed CT image in a 65-year-old male involved in motor vehicle accident ten years back shows a calcified saccular pseudoaneurysm in the aortic isthmus.

Traumatic aortic injury is treated with either open or endovascular repair for descending aortic injuries. The rare patient with ascending injury who survives required open repair via median sternotomy. Repair can be delayed for a short period in hemodynamically stable patients. Subtle injuries usually resolve with conservative management. Traumatic intramural hematomas resolve earlier than spontaneous hematomas since they are periadventitial [17]. Chronic pseudoaneurysms (Figure 11) develop in the isthmus in 2.5% of untreated patients and can calcify and form thrombus with high risk of rupture several years after the initial injury [14].

## 8. Dissection

Aortic dissection is characterized by a tear in the tunica intima of the aorta, with resultant leak of blood into the tunica media and formation of a false lumen. Dissection is the most common cause of acute aortic syndrome and is usually the result of systemic hypertension. Other causes of aortic dissection include Marfan syndrome, connective tissue disorders, bicuspid aortic valve, Turner syndrome, aortic coarctation, aortic aneurysm, aortitis, pregnancy, and cocaine abuse [18].

**Figure 12:**
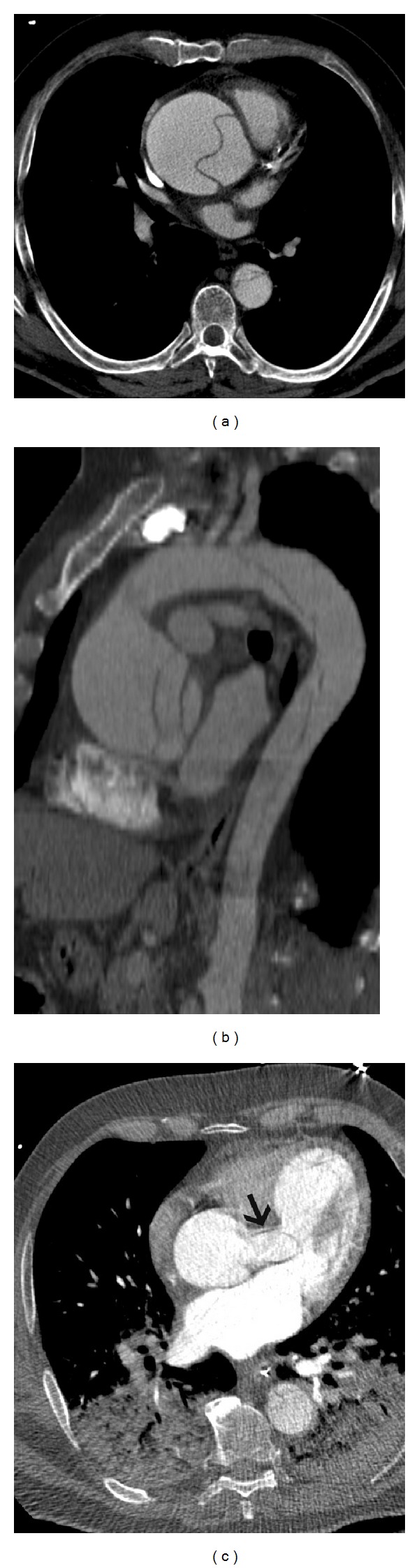
Type  A  dissection—(a) axial postcontrast CT scan in a 56-year-old male shows a dissection flap that involves the ascending aorta and descending thoracic aorta. (b) Sagittal reconstructed post contrast CT image shows the complete dissection flap, beginning from the aortic root and extending across the arch into the descending thoracic aorta. (c) Axial CT scan in another patient shows a Type A dissection flap in the aortic root that is extending into the left coronary artery, which is dilated.

**Figure 13:**
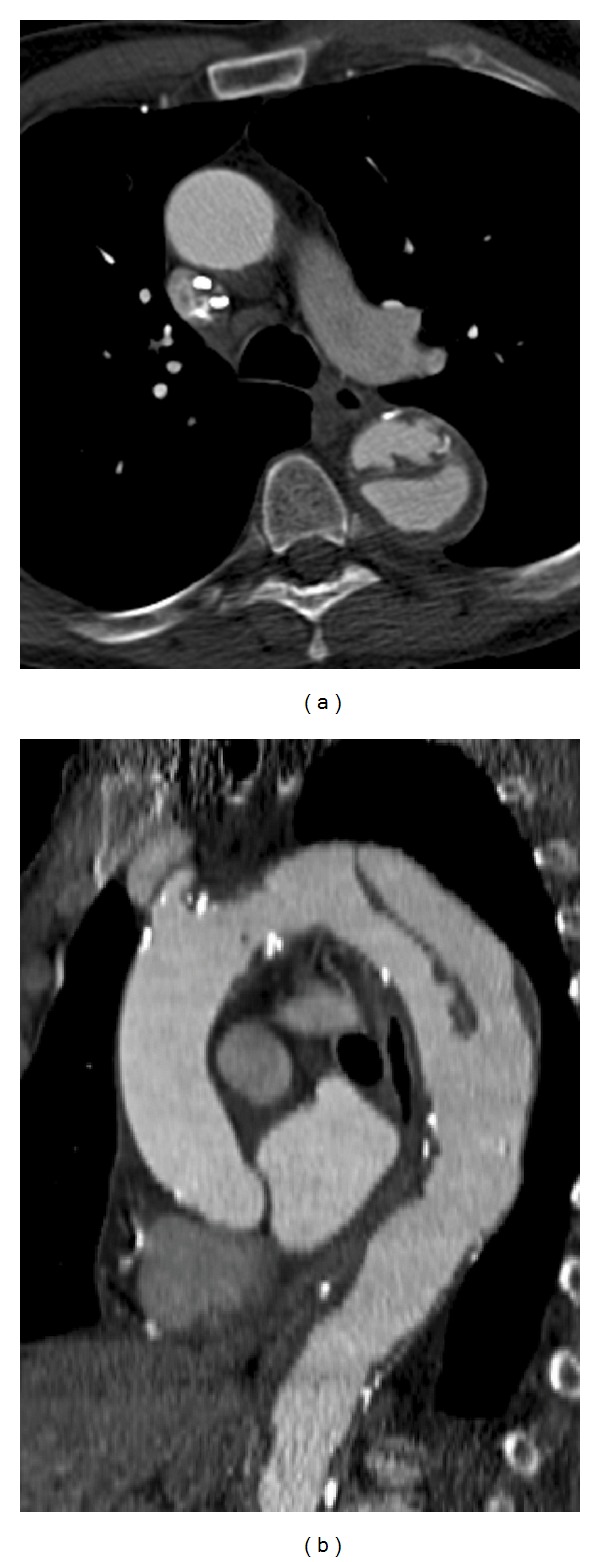
Type  B  dissection—(a) axial CT scan image in a 65-year-old male shows a dissection flap seen only in the descending thoracic aorta but not in the ascending aorta. (b) Sagittal reconstructed post contrast CT image in the same patient shows a dissection flap that involves the arch and proximal descending thoracic aorta, consistent with Type B dissection. The true lumen is anterior and medial, where the false lumen is posterior to lateral.

Aortic dissection is defined as acute when signs and symptoms persist for fewer than two weeks and chronic if they persist more than two weeks. The Stanford classification is used to describe aortic dissection as it dictates treatment. Type A dissections (75%) involve the ascending aorta (Figure 12) and type B dissections (25%) do not involve the ascending aorta (Figure 13). The older DeBakey classification is as follows: type I—tear originates in ascending aorta and propagates to at least arch and often beyond it; type II—tear originates in and is confined to ascending aorta; and type III—tear originates in proximal descending aorta and extends distally to descending thoracic or thoracoabdominal aorta [18].

On unenhanced CT, endoluminal displacement of intimal calcification may be apparent with aortic dissection. Following contrast administration, CT and MRI show an intimal flap separating true from false lumen. The true lumen maintains direct continuity with the undissected proximal normal aorta and is usually smaller than the false lumen. The false lumen can also be identified by the presence of a “cobweb sign,” which describes low attenuation linear foci resulting from residua of incompletely sheared media and the “beak sign,” which describes a wedge of hematoma at the leading edge of the false lumen propagation. With triple barreled dissections, three lumens are present, and either reflects two coexistent dissections on either side of the true lumen or a new third lumen within the wall of false lumen. These most commonly occur with Marfan syndrome. In intimointimal intussception, a circumferential dissection invaginates into itself, with one lumen wrapped around the other, most commonly occurring in the arch.

Thrombus within an aneurysm can mimic aortic dissection. Thrombus has a constant circumferential relationship with the aorta and has irregular borders, and intimal calcification is seen peripheral to the thrombus; however, a dissection has a spiral configuration, smooth inner border, and endoluminal displacement of the intimal calcification by the false lumen [18, 19].

Complications of aortic dissection include aortic rupture, hemothorax, mediastinal hematoma, hemopericardium without or with cardiac tamponade, acute aortic regurgitation, aortic branch vessel obstruction, and end-organ ischemia. Rupture manifests on CT as irregular aortic wall with active contrast extravasation with high attenuation collection(s) in the pericardium, left extrapleural space, or mediastinum. Rarely, mediastinal blood can seep through the adventitial space between the aorta and pulmonary artery and eventually dissect the pulmonary artery. Blood seeping from the posterior wall of the aorta can also reach the lung tracking along the bronchovascular sheaths.

End-organ ischemia results from extension of the dissection flap into the artery supplying the organ or thrombosis of a true or false lumen that supplies the visceral artery in question. Arterial obstruction can be either dynamic with prolapse of the intimal flap covering the false lumen or static resulting from compression of the artery by the expanding false lumen or adjacent hematoma. Obstruction of the carotid arteries occurs in 5–10% of patients with acute aortic dissection, and the superior and inferior mesenteric and renal arteries are affected in 27% of patients. A small true lumen with concavity to the false lumen indicates low pressure and possibility of ischemia. Static obstruction is relieved by placing a stent, but dynamic obstruction requires fenestration to relieve pressure in false lumen [18].

Type A dissections require prompt surgical treatment because of the high risk of rupture and cardiac tamponade. Uncomplicated type B dissections are usually treated medically and require serial followup with CT or MRI. Type B dissections with complications require endovascular or surgical repair.

## 9. Intramural Hematoma

Intramural hematoma (IMH) is characterized by spontaneous hemorrhage into the tunica media of the aorta, most commonly the result of rupture of the vasa vasorum and less commonly the result of penetrating atherosclerotic ulcer or trauma. Hypertension, atherosclerosis, and advanced age are the major risk factors for IMH. Although no intimal tear is present, the tunica media is weakened and the clinical presentation and course are similar to acute aortic dissection. IMH accounts for 10–20% of patients with acute aortic syndrome. Like aortic dissection, IMH is classified into Type A or B with 70% involving the ascending and proximal descending segments [11].

**Figure 14:**
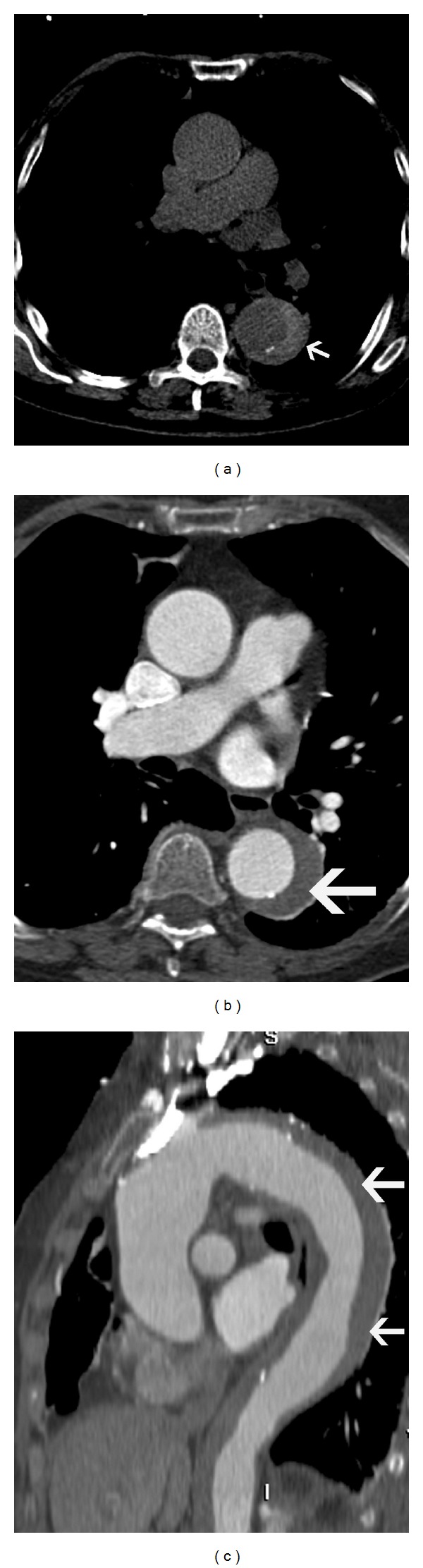
Intramural  hematoma—(a) noncontrast axial CT scan of a 67-year-old male with acute chest pain shows a high density collection (arrow) in the periphery of the descending aorta, which is internally displacing intimal calcification. (b) Axial postcontrast CT scan of the same patient shows the hypodense collection (arrow) in the periphery of the contrast opacified aorta. Although it appear hypodense, the mean attenuation value was 80 HU, which is higher than that for a thrombus. Also, the intimal calcification goes against a luminal thrombus. (c) Sagittal postcontrast CT scan shows the craniocaudal extent of the intramural hematoma (arrows).

On unenhanced CT, intramural hematoma manifests as a crescent of high attenuation within the aortic wall, often extending circumferentially and narrowing the lumen and sometimes displacing intimal calcifications toward the lumen (Figure 14(a)). On contrast-enhanced CT, the hematoma shows no enhancement and maintains a constant circumferential relationship with the aortic wall (Figures 14(b) and 14(c)). The intramural hematoma can extend along the aorta, rupture or cause cerebral, mesenteric vascular, or renal ischemia. On spin echo MRI images, intramural hematoma shows intermediate signal intensity during the acute phase because of the presence of oxyhemoglobin and high signal intensity during the subacute stage because of the presence of methemoglobin [11].

The relationship between IMH and dissection is not well established. Some studies have shown progression of IMH to dissection, implying that IMH is an early stage or variant of dissection. In one study, 8% of patients with aortic dissection were shown to have had a previous IMH [20]. Reported features associated with progression to aortic dissection include involvement of the ascending aorta (type A), thick hematoma (>10 mm), dilated aorta (>5 cm), mural ulcer, compressed aortic lumen, focal contrast enhancement (possibly representing an intimal microtear), pericardial effusion, and pleural effusion [18]. Similar to aortic dissection, type A IMH usually requires open surgical repair, while type B IMH can often be managed medically except in the presence of aneurysm, progression, dissection, impending rupture, or end-organ ischemia, with any of which open or endovascular repair is performed. Serial follow-up CT or MRI examinations are indicated for patients who are followed conservatively [21].

## 10. Penetrating Atherosclerotic Ulcer

Penetrating atherosclerotic ulcer is characterized by ulceration of the aortic wall, usually by atherosclerosis, most commonly develops in the mid descending aorta or arch, and very rarely develops in the ascending aorta, which is generally protected from atherosclerosis by rapid blood flow. A penetrating atherosclerotic ulcer begins as an atherosclerotic intimal ulcer (stage I), subsequently evolving into an intimal tear (stage II), followed by hemorrhage into the media (stage III), and finally resulting in a full thickness penetration of the aortic wall (stage IV). They usually present in older patients and can be solitary or multiple. Patients usually present with acute chest pain similar to aortic dissection.

**Figure 15:**
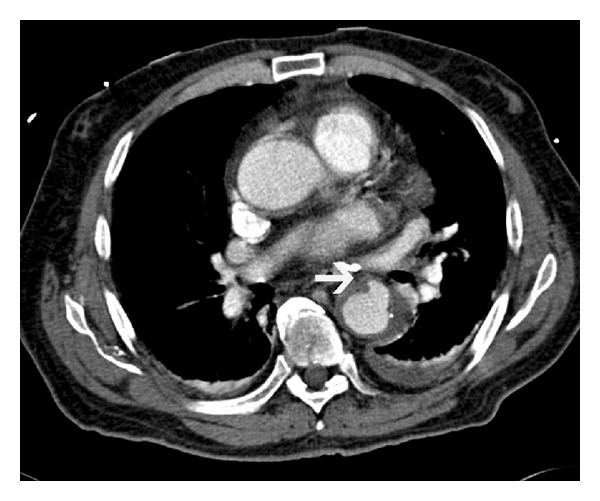
Penetrating atherosclerotic ulcer—axial postcontrast CT scan in a 48-year-old male with acute chest pain shows contrast outpouching from the anterior aspect of descending thoracic aorta (arrow), consistent with a penetrating atherosclerotic ulcer.

Penetrating atherosclerotic ulcer is difficult to detect on unenhanced CT. Indirect findings such as intramural hematoma, atherosclerosis, and displaced intimal calcification may be present. Contrast-enhanced CT shows a collection of contrast-enhanced blood outside the normal aortic lumen (Figure 15). The aortic wall may be thickened or may show contrast enhancement. A penetrating ulcer should be distinguished from an atherosclerotic ulcer, which is confined to the intima, lacks intramural hematoma, and results in no symptoms. Saccular aneurysm, dissection, distal embolization, and rupture can ensue.

Penetrating ulcer is usually managed medically, but open or endovascular repair is performed if patients become hemodynamically unstable or have persistent or worsening pain or have rapid ulcer enlargement, aortic rupture, or distal embolization of thrombus. Surgery may be technically difficult since the aortic wall is damaged by the ulcer, with high morbidity and mortality [18].

## 11. Rupture 

Rupture of an aneurysm, dissection, or penetrating ulcer can be contained or can be free when it extends into the mediastinum, pericardium, pleura or extrapleural space, esophagus, or bronchus. Ruptured aneurysms are often diagnosed clinically, but if imaging is required, CT is the modality of choice because of rapid acquisition, wide spread availability, and high diagnostic accuracy [11]. Unenhanced CT shows the hematoma extending from the aneurysm, dissection, ulcer, or IMH. Contrast-enhanced CT shows active extravasation into the adjacent tissues. High attenuation collections can be seen in the pleura, extrapleural space, pericardium, or mediastinum. Aortic rupture into the esophagus or a central airway is often fatal. With aortoesophageal fistula, contrast accumulates in the esophagus and air may be present in the aorta. Mediastinal hematoma is usually present. Aortobronchial fistulas most commonly develop between the descending aorta and left main bronchus. The exact site of communication is difficult to identify, but pulmonary consolidation develops because of blood filling the affected airways [22].

**Figure 16:**
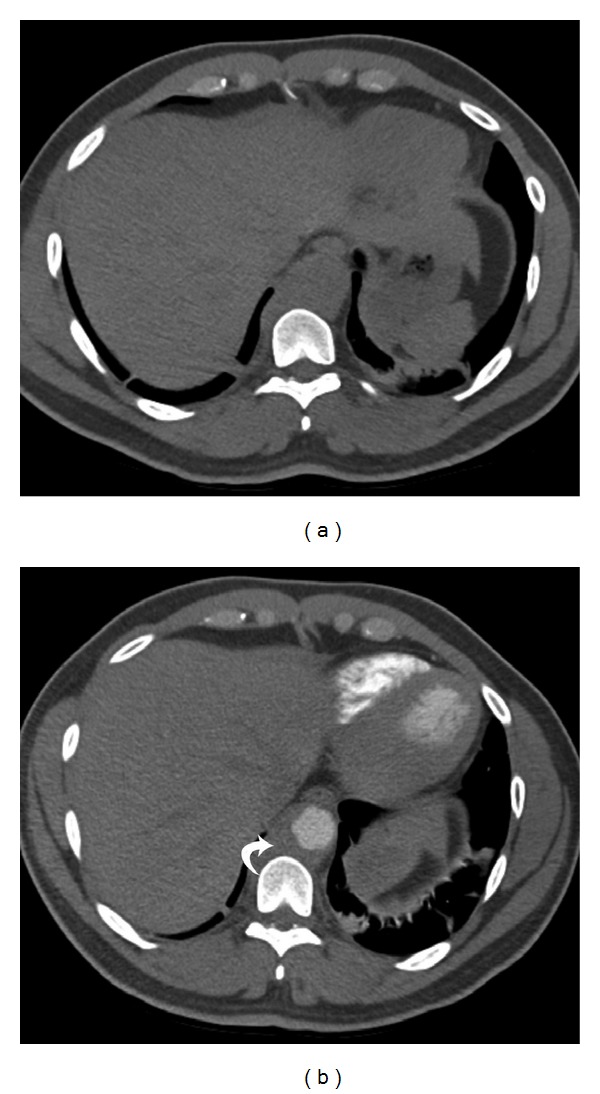
Contained rupture—(a) noncontrast CT scan in a patient presenting with acute chest pain shows nonspecific soft tissue adjacent to descending thoracic aorta. (b) Contrast enhanced CT at the same level shows lack of distinction between the posterior wall of the aorta and the adjacent vertebral body, the “draped aorta” sign (curved arrow), which is indicative of a contained rupture.

Impending rupture manifests on CT by the presence of a high attenuation “crescent” within mural thrombus of an aneurysm. Contained rupture is characterized lack of distinction between the posterior wall of the aorta and adjacent structures such as the vertebrae a result of contained rupture of the posterior aortic wall, a finding referred to as the “draped aorta” sign [14] (Figure 16).

## 12. Mycotic Aneurysm

**Figure 17:**
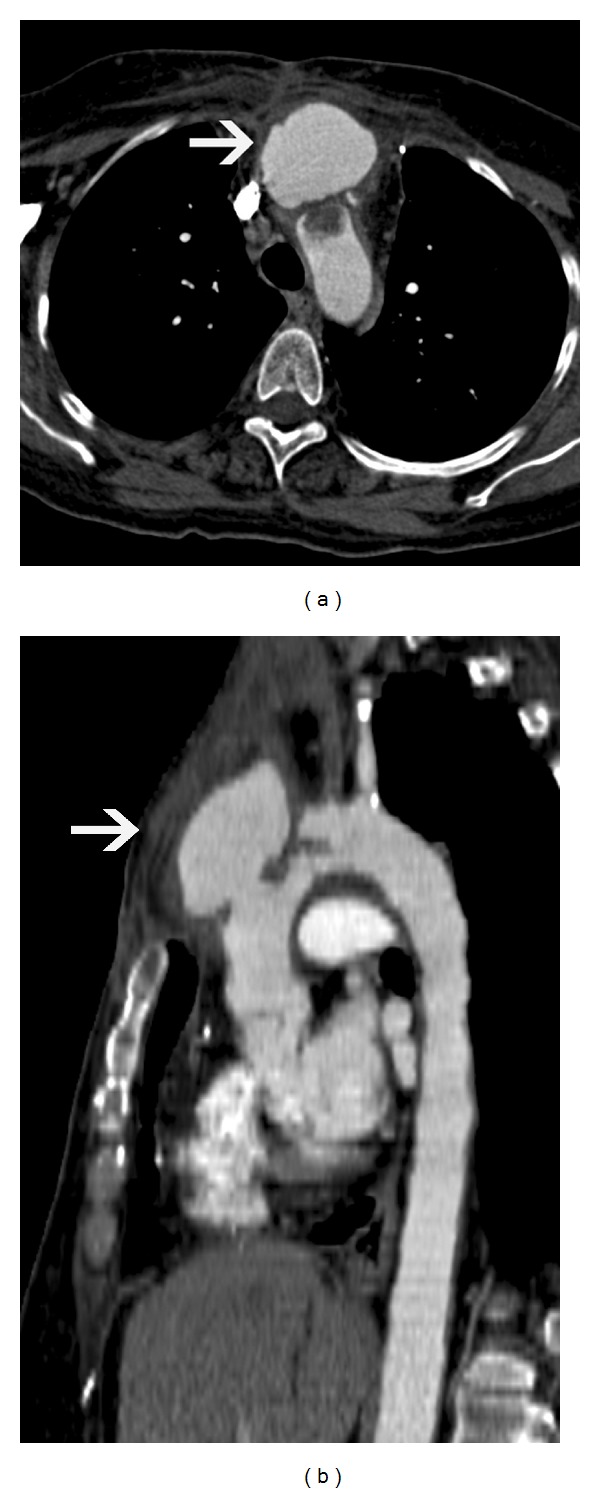
Mycotic  aneurysm—(a) axial postcontrast CT scan of a 72-year-old male shows a large saccular pseudoaneurysm (arrow) arising from the anterior aspect of the distal ascending aorta, which is anteriorly eroding the sternum. (b) Sagittal postcontrast CT scan in the same patient shows the focal mycotic pseudoaneurysm (arrow) that is arising from the anterior aspect of ascending aorta and eroding the anterior sternum.

Mycotic aneurysm is infectious aneurysm caused by nonsyphilitic organisms such as nonhemolytic *Streptococcus*, *Staphylococcus*, *S. pneumoniae*, *Gonococcus*, and *Salmonella*. Although the aortic intima is typically resistant to infection, weakening from atherosclerosis, bacterial endocarditis, drug abuse, and trauma predispose the aorta to infection. Mycotic aneurysms are more common in the ascending aorta because of their proximity to regions affected by endocarditis (Figure 17). Mycotic aneurysms can be multiple and are usually saccular with a wide neck [14].

## 13. Aortitis

Aortitis (vasculitis) is characterized by presence of leucocytes in the aorta wall, with reactive damage to mural structures. Takayasu arteritis and giant cell arteritis are the most common vasculitides to affect the aorta.

Takayasu arteritis is an idiopathic arteritis that is more common in women, particularly Asians. It destroys the arterial media, resulting in aneurysms and rupture. Clinically, it has an early systemic phase with patients having nonspecific constitutional symptoms and a late occlusive phase in which patients may present with signs and symptoms of vascular disease. Takayasu arteritis is classified into four types:   type  1—classic pulseless; type  2—mixed; type  3—atypical coarctation; or type  4—dilated. It typically affects the aorta and its primary branches and can involve a focal region of the aorta or the entire vessel. Takayasu arteritis can be aneurysmal or occlusive. Based on the distribution of vascular involvement, Takayasu can be classified using the Numano system as per American College of Rheumatology guidelines: type  I—involvement of aortic arch and branch vessels; type IIa—involvement of the ascending aorta or arch, with or without branch vessel involvement; type IIb—involvement of the descending thoracic aorta with or without involvement of the ascending aorta or arch with branches; type III—involvement of the descending thoracic aorta and abdominal aorta with or without renal artery involvement; type  IV—involvement of only abdominal aorta with or without renal artery involvement; type  V—involvement of the entire aorta with branch vessel involvement. Coronary arteries (C+) and pulmonary arteries (P+) may be involved in any type [23].

**Figure 18:**
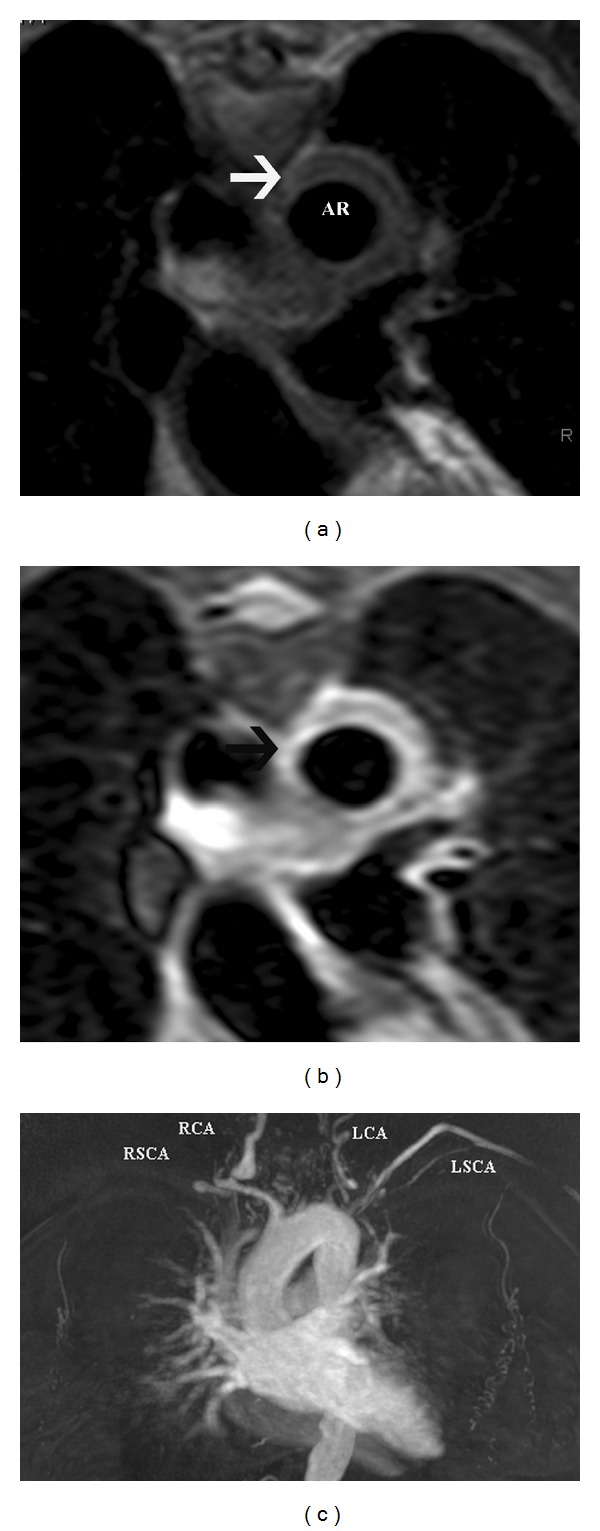
Takayasu   arteritis—(a) axial T2-weighted double inversion recovery black blood image in a 31-year-old female patient with Takayasu arteritis shows severe circumferential thickening of the aortic arch (arrows). (b) T2-weighted triple inversion recovery black blood image in the same patient with Takayasu arteritis shows very high signal of the thickened aortic arch (arrows), which is consistent with edema, indicative of active arteritis. (c) 3D contrast enhanced MR angiography images of the arch demonstrate severe stenosis of the left common carotid (LCA), right common carotid artery (RCA), and proximal left subclavian artery (LSCA) with occlusion of the right subclavian artery (RSCA).

CT and MRI can show thickening of the vessel wall and mural contrast enhancement in early stages; stenoses, occlusion, and aneurysms characterize the later stages [24]. MRI shows wall thickening and high signal intensity on T2-weighted STIR images due to edema in acute stages (Figure 18). Thickened aortic cusps and pericardial effusion or thickening may also be present. Delayed contrast enhancement has been described as a feature of active disease [25].

Takayasu arteritis is usually treated with high dose steroids. Stenoses and occlusion often require endovascular or open surgical therapy.

**Figure 19:**
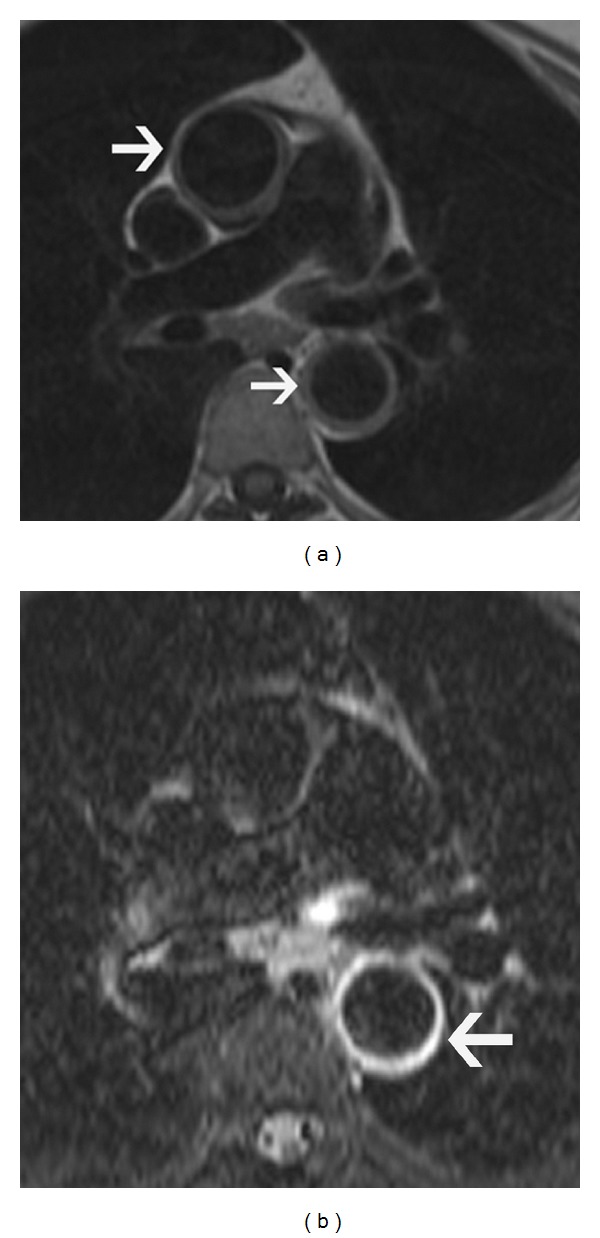
Giant  cell  arteritis—(a) axial T2-weighted double inversion recovery black blood image in a 68-year-old female patient with giant cell arteritis shows mild circumferential thickening of the ascending and descending aorta (arrow). (b) T2-weighted triple inversion recovery black blood image in the same patient with giant cell arteritis shows very minimal high signal of the descending aorta (arrow), which is consistent with mild active arteritis.

*Giant cell (temporal) arteritis* is a chronic vasculitis of large and medium size vessels that typically affects the temporal arteries and is associated with polymyalgia rheumatica. The large vessel type of giant cell arteritis affects the aorta and its branches, particularly the subclavian arteries (Figure 19). Although prognosis is similar to that of nonlarge vessel type, the risk of dissection is greater and is associated with a poor outcome [26, 27].

## 14. Marfan Syndrome

Marfan syndrome is an autosomal dominant inherited connective tissue disorder caused by mutations in the fibrillin (FBN1) gene. It has a prevalence of 2-3 per 10000 [28]. Approximately 25–30% of these cases are sporadic. Fibrillin is a major component of microfibrils, which connect elastic laminae to endothelial and smooth muscle cells and are responsible for the structural integrity and elastic tension of the vascular wall. Mutations result in elastic fiber fragmentation, structural disintegration, and aneurysms. Pathologically, elastic fibers are degenerated and cystic necrosis of smooth muscle cells is present in the medial layer of arteries, resulting in increased stiffness and loss of distensibility.

Annuloaortic ectasia with or without aortic regurgitation, aortic dissection, pulmonary artery dilation, and mitral valve prolapse are the common complications of Marfan syndrome. Aneurysms in Marfan syndrome occur at a younger age, enlarge faster, and usually lack calcification or thrombus. Segments other than ascending aorta are less commonly affected, although the distal aorta can be involved following ascending aortic repair. The rapid onset and progression distinguish it from idiopathic medial degeneration [14]. Dissection is common and can be recurrent or triple barreled, the latter of which is prone to rupture. Aneurysms and dissections in patients with Marfan syndrome are repaired at a size smaller than conventional aneurysms. Musculoskeletal manifestations are scoliosis, pectus excavatum, pectus carinatium, arachnodactyly, and acetabular protrusion. Dural ectasia is a characteristic neurological manifestation. Pulmonary cysts and ocular involvement are also seen. Diagnosis is made by using the Ghent classification, depending on major and minor criteria involving multiple systems. Aortic dissection, cardiac failure, and valvular disease are the most common causes of death [28].

## 15. Loeys-Dietz Syndrome

Loeys-Dietz syndrome (LDS) is caused by mutations in genes encoding type I or II transforming growth factor *β* (TGF-*β*). In type I, craniofacial (hypertelorism, bifid uvula, cleft palate, craniosynostosis) and vascular (arterial aneurysms and tortuosity) abnormalities are present. In type II, bifid uvula occurs without other craniofacial abnormalities. Death occurs at later age in type II than type I. Aneurysms of the aortic root develop in 98% of patients because of reduced elastin.

The vascular manifestations of LDS are more severe than those of Marfan syndrome. Aneurysms occur at a younger age, involve vessels other than aorta (92%), and rupture at smaller diameters. Dissections are more frequent, occur at a younger age, and rupture at smaller diameters. In addition, the involved vessels are more tortuous. Pulmonary artery dilation occurs in both LDS and Marfan. Congenital cardiac anomalies, such as patent ductus arteriosus, bicuspid aortic valve, mitral valve prolapse, and atrial septal defect, may be associated. The type II Loeys-Dietz syndrome can mimic Ehlers-Danlos syndrome (EDS) with spontaneous rupture of arteries, the spleen, uterus, and bowel. However, unlike the EDS, the vascular wall is not friable. Surgery is performed in adults when the aorta is 4 cm or larger in diameter and in children when the aortic root *z* score and annulus are >1.8 cm. Surveillance imaging is usually performed annually [29].

## 16. Vascular Ehlers-Danlos Syndrome

Ehlers-Danlos syndrome is a heterogeneous group of connective tissue disorders, with various inheritance patterns. The various types include—classic; hypermobility; vascular; kyphoscoliosis; arthrochalasia; and dermatosparaxis. The vascular type (EDS IV) is rare (<4%) and is caused by mutation of the type III procollagen (COL3A1) gene that results in fragile medium and large arteries. Vascular, intestinal, and uterine rupture can occur. Excessive bruising, thin and translucent skin, and characteristic facial appearance (thin, pinched nose, thin lips, and prominent bones and eyes) may be apparent. Imaging findings include ectasia, aneurysm, dissection, occlusion, rupture, and fistulas of large and medium arteries. EDS IV affects the abdominal visceral branches, iliac arteries, and thoracoabdominal aorta more commonly than the lower limb, carotid, vertebral, subclavian arteries, and pulmonary and cerebral arteries. Multiple vascular segments are involved at a young age. Vascular changes are progressive, necessitating frequent followup. Due to friable vessel walls, surgery is performed only with rupture or impending rupture. Invasive angiography and interventional procedures are usually avoided. EDS IV has the worst prognosis among all types of EDS due to hemorrhage and infarcts involving the cerebral, renal, and splenic vessels, which are the most common causes of death [30].

## 17. Conclusion

CT and MRI are the ideal noninvasive imaging examinations for the evaluation of thoracic aortic abnormalities. Knowledge of the imaging appearances of these lesions enables preoperative or interventional assessment and postprocedural followup for detection of complications.
